# Citrate-Coated Magnetic Polyethyleneimine Composites for Plasmid DNA Delivery into Glioblastoma †

**DOI:** 10.3390/polym13142228

**Published:** 2021-07-06

**Authors:** Ken Cham-Fai Leung, Kathy W. Y. Sham, Josie M. Y. Lai, Yi-Xiang J. Wang, Chi-Hin Wong, Christopher H. K. Cheng

**Affiliations:** 1 State Key Laboratory of Environmental and Biological Analysis, Department of Chemistry, The Hong Kong Baptist University, Kowloon Tong, KLN, Hong Kong, China; 12467375@life.hkbu.edu.hk; 2 School of Biomedical Sciences, The Chinese University of Hong Kong, Shatin, NT, Hong Kong, China; kathysera@cuhk.edu.hk (K.W.Y.S.); josiepig@cuhk.edu.hk (J.M.Y.L.); 3 Department of Imaging and Interventional Radiology, Prince of Wales Hospital, The Chinese University of Hong Kong, Shatin, NT, Hong Kong, China; yixiang_wang@cuhk.edu.hk

**Keywords:** citrate, gene delivery, magnetic nanoparticle, nanostructure, polyethyleneimine

## Abstract

Several ternary composites that are based on branched polyethyleneimine (bPEI 25 kDa, polydispersity 2.5, 0.1 or 0.2 ng), citrate-coated ultrasmall superparamagnetic iron oxide nanoparticles (citrate-NPs, 8–10 nm, 0.1, 1.0, or 2.5 µg), and reporter circular plasmid DNA pEGFP-C1 or pRL-CMV (pDNA 0.5 µg) were studied for optimization of the best composite for transfection into glioblastoma U87MG or U138MG cells. The efficiency in terms of citrate-NP and plasmid DNA gene delivery with the ternary composites could be altered by tuning the bPEI/citrate-NP ratios in the polymer composites, which were characterized by Prussian blue staining, in vitro magnetic resonance imaging as well as green fluorescence protein and luciferase expression. Among the composites prepared, 0.2 ng bPEI/0.5 μg pDNA/1.0 µg citrate-NP ternary composite possessed the best cellular uptake efficiency. Composite comprising 0.1 ng bPEI/0.5 μg pDNA/0.1 μg citrate-NP gave the optimal efficiency for the cellular uptake of the two plasmid DNAs to the nucleus. The best working bPEI concentration range should not exceed 0.2 ng/well to achieve a relatively low cytotoxicity.

## 1. Introduction

**Scheme 1:**
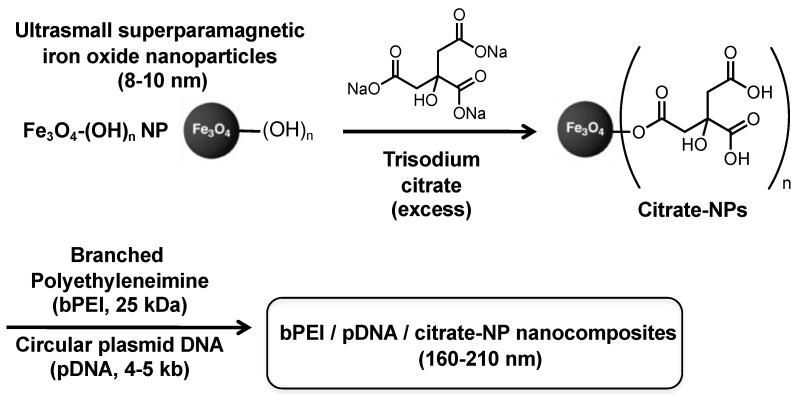
Schematic illustration of the preparation of the citrate-NPs and the nanocomposites.

Novel theranostic nanomaterials [1,2,3,4] had been demonstrated for their fascinating properties in the co-delivery of genes and drugs. Their intrinsic properties as probes for various imaging techniques had also been developed rapidly for targeted brain cancer diagnosis and treatment [5,6,7]. Ultrasmall iron oxide nanoparticles (USIO NPs), by way of examples, offered properties including Fenton reactions [8], magnetic resonance imaging (MRI), magnetic targeting, cellular tracking, drug, and gene delivery to specific target site(s) [9,10,11,12,13,14,15,16,17,18]. On the other hand, polyethylenimine (PEI) polymers with branched structures had been investigating their properties to deliver genes and drugs with enhanced transfection and targeting efficiencies as well as with minimal cytotoxicities [19,20,21,22,23,24]. USIO composite materials with branched PEIs with alginate or deferoxamine had been demonstrated [25] with reduced cytotoxicities and the properties of different coatings. USIO-NPs that were coated with negatively charged citrate derivatives were less studied for biomedical purposes [26,27,28], partly because of the difficulty of transfecting them and drugs to the brain and many other organs without severe agglomeration. It is known that plasmid DNA-based gene therapy by direct delivery is less efficient [29,30,31,32]. We aimed to investigate the potential of our theranostic nanomaterials to deliver these kinds of materials with improved and/or promising transfection (delivery) efficiencies. Two expression plasmids encoding two separate reporter genes (EGFP and *Renilla* luciferase) were used to evaluate the transfection efficiency of plasmid DNA into glioblastoma cells. For the purpose of delivering designed plasmid DNAs towards a glioblastoma cell line U87MG transfection with much enhanced cellular uptake efficiency, we report herein the preparation of citrate-coated Fe_3_O_4_ USIO NPs (citrate-NPs, 8–10 nm), hybridizing with circular plasmid DNAs (pDNAs, pEGFP-C1 and pRL-CMV, 4 kb), and branched PEI (bPEI, MW 25 kDa, polydispersity 2.5) to furnish the ternary composites (Scheme 1) [33,34,35]. These composites had been for studied for MRI, fluorescence imaging and cytotoxicities. Any reduction or enhancement of the plasmid DNA uptake by the composites can be estimated by using two individual detection methods. Circular plasmid DNA pEGFP-C1 (4.7 kb) encoded with a red-shifted variant of wild-type green fluorescence protein (GFP) in mammalian cells as well as the pRL-CMV (4.0 kb) encoded with a *Renilla* luciferase in various cell types were employed. A variety of cell types and cell lines could be optimized because the reporter genes could be expressed as luminescence or fluorescence intensities. These intensities were directly proportional to the amounts of the luciferase or GPF in the cells. By the strong, enhanced and constitutive expression of the reporter genes, the signals can be easily detected. It was envisaged that after the uptake of the composites into the cells, the NPs in the composites could be cleaved thereby generating the negative (dark) MRI contrast signal. On the other hand, the plasmid DNA of the composites could further be translocated into the nucleus for expressing the reporter genes.

## 2. Materials and Methods

All reactions were carried out under high purity (99.9%) nitrogen atmosphere. Deionized water was obtained from Barnstead RO pure system. All solvents were bubbled with high purity nitrogen for at least 30 min before use. Ultrasmall superparamagnetic iron oxide nanoparticles with average diameter range of 8–10 nm were synthesized according to the literature procedures [36,37,38], which contain hydroxyl functional group as the periphery, i.e., Fe_3_O_4_−(OH)_n_. Citrate-coated Fe_3_O_4_ USIO NPs (citrate-NPs) were prepared as follows. In particular, Fe_3_O_4_−(OH)_n_ (66 mg) NPs were treated with an excess of trisodium citrate (1.01 g, Sigma-Aldrich, St. Louis, MO, USA) in water (25 mL) at 90 °C with mechanical stirring for an hour. The citrate-NPs were magnetically separated and washed repeatedly with water and ethanol. The residue was dried in high vacuum overnight to create the citrate-NPs. FT-IR stretching frequencies: 3443 cm^−1^ (O–H), 1624 cm^−1^ (C = O), 1053 cm^−1^ (C–OH) and 580 cm^−1^ (Fe–O). The size of the ternary complex was determined by the dynamic light scattering (DLS) to be 160–210 nm. Furthermore, stability test of the ternary complex was performed for a week, revealing no obvious size change.

Both U87MG and U138MG glioblastoma cell lines were acquired from the American Type Culture Collection. Cells were cultured with α-MEM (Thermo Fisher Scientific, Waltham, MA USA) containing 10% fetal bovine serum, 100 µg/mL streptomycin and 100 U/mL penicillin, in a humidified 5% CO_2_ atmosphere at 37 °C.

All circular plasmid DNAs (pDNAs) were prepared using the QIAprep Spin Miniprep Kit (QIAGEN) with an A_260_/A_280_ ratio larger than 1.8. For the synthesis of composites, a stock solution of branched PEI (25 kDa, polydispersity 2.5, Sigma-Aldrich, St. Louis, MO, USA) was prepared with a concentration of 10 ng/µL in water. By serial dilutions, the solutions of branched PEI with different concentrations were added to the culture medium containing the plasmid DNA. After incubation for 30 min, pre-ultrasonicated, citrate-NPs of known particle and iron concentrations (ICP-MS) in water were added to the mixture, gently mixed and incubated for further 30 min to obtain the composites.

To evaluate the cytotoxicities of the magnetic composites, 5000 cells were seeded onto each well of 96-well plates for methylthiazolyldiphenyl-tetrazolium bromide (MTT) assay. On the other hand, 50,000 cells were seeded onto each well of 24-well plates for luciferase assay and fluorescence microscopy. Next day, the culture medium was replaced with the serum-free α-MEM containing different composites. After incubation of the composites for 5 h, the medium was aspirated and refreshed with complete α-MEM. The cells were incubated for further 24 h at 37 °C for subsequent assays (*n* = 2).

The green fluorescence in glioblastoma cells was visualized by a Nikon TE2000 fluorescence microscope and luminescence was detected by a luminometer (GloMax 20/20 Luminometer, Promega, Madison, WI, USA). Luciferase expression of different composites in glioblastoma cells. *Renilla* luciferase reporter plasmid pRL-CMV was mixed with different amounts of bPEI and citrate-NP to form the composites. The amount of pRL-CMV of all composites was fixed at 0.5 μg/well. These composites were incubated with U87MG and U138MG cells for 5 h. Twenty-four hours after transfection, cells were harvested and lysed. *Renilla* activities (RLU) were then measured and normalized against total cellular protein per well.

The cells were washed with PBS to remove any free composites. Cells were then fixed using paraformaldehyde (4%) for 40 min. Subsequently, cells were washed with PBS and incubated with freshly prepared Perls’ reagent (4% potassium ferrocyanide and 12% HCl, 1:1 *v*/*v*) for 30 min. Cells were washed with PBS three times, counterstained with neutral red (0.02%), and subsequently observed by an inverted bright-field optical microscope (Nikon TE2000).

The viability of cells incubated with different composites was estimated by MTT assay in glioblastoma cells. Ten microliters of 5 mg/mL MTT solution was added into each well. After incubation for 3 h, the medium was removed, and formazan crystals were dissolved in dimethyl sulfoxide (150 µL) for 10 min on a shaker. A small round disc-like magnet was placed under the plate to the bottom of the well and to attract the magnetic composite-uptaken cells. After that, 100 µL of the supernatant was transferred to another 96-well plate. Absorbance of each well was measured on a microplate reader (Bio-Rad, Model 3550) at a wavelength of 540 nm. The relative cell viability (%) for each sample which is related to the control well, was calculated.

For in vitro MRI, after washing with PBS, the cells were trypsinised and counted. Different numbers (12.5k, 25k, 50k, 100k, 150k, and 300k) of cells were placed in an Eppendorf tube (1.5 mL) separately. After centrifugation at 3000× *g* for 5 min, the Eppendorf tubes were placed perpendicularly to the main magnetic induction field (*B*_0_) in a 20 × 12 × 8 cm^3^ water bath. MRI was performed with a 3.0-T clinical whole-body magnetic resonance unit (Achieva, Philips Medical Systems) using a transmit–receive head coil. The magnetic resonance sequence was a two-dimensional gradient-echo sequence with TR = 400 ms, TE = 48 ms, flip angle = 18°, matrix = 512 × 256, resolution = 0.45 × 0.45 mm, slice thickness = 2 mm, and number of excitations = 2. Sagittal images were obtained through the central section of the bottom tips of the Eppendorf tubes. The areas of signal void at the bottom of the Eppendorf tubes due to the citrate-NP-containing composites uptaken into U87MG cells from which the NPs is the MRI-responsive contrast agent. The *T*_2_ relaxation times were measured by using a standard Carr–Purcell–Meiboom–Gill pulse sequence with the following parameters: repetition time TR = 2000 ms, echo time TE range = 30–960 ms, 32 echoes, field-of-view = 134 × 67 mm^2^, matrix = 128 × 64, slice thickness = 5 mm, number of excitations = 3. *T*_2_ relaxation times were calculated by fitting the logarithmic region of interest signal amplitudes versus TE. The *T*_2_ relaxivities (*r*_2_) were determined by a linear fit of the inverse relaxation times as a function of the iron concentrations used.

## 3. Results

Both USIO nanoparticles with negatively charged citrate coating as well as the negatively charged plasmid DNAs could be noncovalently self-assembled [39] to the positively charged branched PEI polymers to furnish the ternary composites (160–210 nm), stabilized by mainly multiple ionic (CO_2_^−^···NH^+^) charge attractions between the carboxylic acid group of the citrate and the amine group of the PEI. The polymeric nature of the materials provided multivalent, strong interactions to furnish the stable composites. The morphology and surface functional groups of the composites were characterized by transmission electron microscopy (TEM) and Infrared (IR) absorption spectroscopy, which had been reported in the literature [19,30,31]. The as-prepared citrate-NPs had a narrow size distribution (8–10 nm). IR spectra of these NPs reveal their functional group characteristic signals at 580 cm^−1^ for the Fe–O, 1053 cm^−1^ for the C–OH, 1624 cm^−1^ for the carbonyl C=O and 3443 cm^−1^ for the O–H moieties.

The size of the ternary complex was determined by DLS in buffer to be 160–210 nm. Stability test of the ternary complex was performed for a week, revealing no obvious size change (180 ± 20 nm) by DLS.

**Figure 1:**
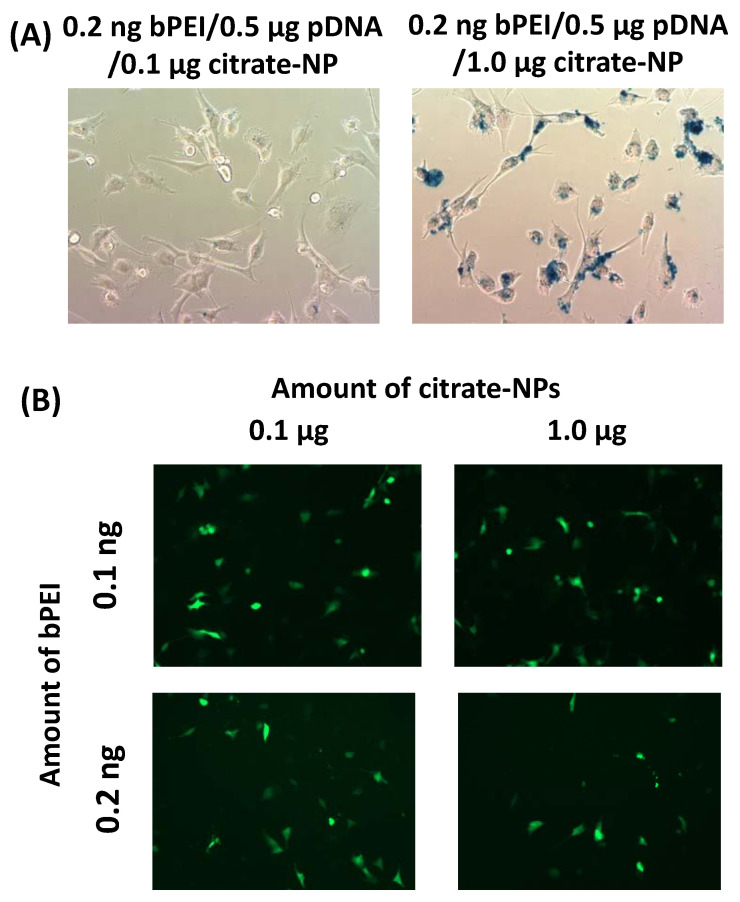
(**A**) Prussian staining images of two different composites 0.2 ng branched bPEI/0.5 μg pEGFP-C1 pDNA/0.1 μg citrate-NP and 0.2 ng branched bPEI/0.5 μg pEGFP-C1 pDNA/1.0 μg citrate-NP 24 h after incubation with each composite for 5 h in U87MG cells. (**B**) GFP fluorescence images of four different composites with a fixed amount of 0.5 μg pEGFP-C1 pDNA 24 h after incubation with each composite for 5 h in U87MG cells.

Composites with varying amounts of citrate-NPs (0.1 to 1.0 μg/well), together with fixed amounts of bPEI (0.2 ng/well) and pDNA (0.5 μg/well) had been separately internalized with the U87MG cells. The cellular uptake efficiencies generally increased when the citrate-NP concentration increased from 0.1 and 1.0 μg/well, as indicated by the Prussian Blue staining of the citrate-NPs (Figure 1A). The typical GFP green, fluorescent images of the U87MG cells which had been separately internalized with four different composites with different amounts of bPEI (0.1 and 0.2 ng/well) and citrate-NPs (0.1 and 1.0 μg/well) are shown in Figure 1B. All four composites showed cellular uptake efficiency with significant green fluorescence observed with the U87MG cells.

**Figure 2:**
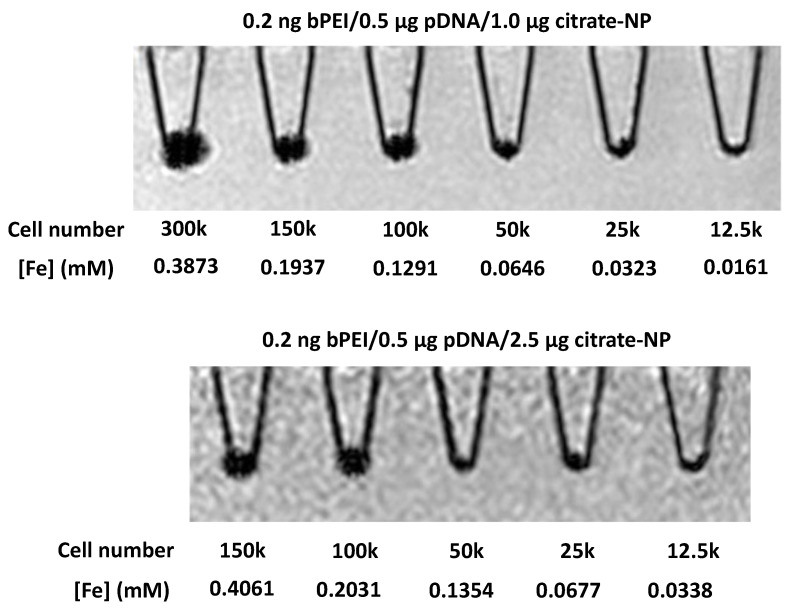
Gradient echo in vitro MRI images of the two composites (0.2 ng branched bPEI/0.5 μg pEGFP-C1 pDNA/1.0 μg citrate-NP and 0.2 ng branched bPEI/0.5 μg pEGFP-C1 pDNA/2.5 μg citrate-NP)-transfected U87MG cells in Eppendorf tubes with culture medium.

U87MG cells that were incubated separately with two different composites of citrate-NP (1.0 and 2.5 μg/well) with fixed bPEI (0.2 ng/well) and pDNA (0.5 μg/well) amounts were analysed by in vitro MRI. Substantial negative (dark) contrast MRI signals with a ballooning effect were observed as shown in Figure 2 with the cells that were centrifuged at the bottom of the Eppendorf tubes. Increasing number of ternary composite-incubated cells gave a larger MRI dark contrast signal. The MRI contrast signal with a cell number of 12.5k was small and yet still observable. The iron concentrations [Fe] of all cell samples were determined by the inductively coupled plasma mass spectrometry (ICP-MS). By measuring the pixels of the dark contrasts related to the iron concentrations, the in vitro T_2_ relaxivities (*r*_2_) of the two composites (0.2 ng bPEI/0.5 μg pEGFP-C1 pDNA/1.0 μg citrate-NP and 0.2 ng bPEI/0.5 μg pEGFP-C1 pDNA/2.5 μg citrate-NP) were determined to be 45.9 and 43.9 s^−1^ µM^−1^ Fe, respectively. Comparatively, the composites with 1.0 μg/well citrate-NPs possessed the highest MRI signal intensities than that of the 2.5 μg/well.

**Figure 3:**
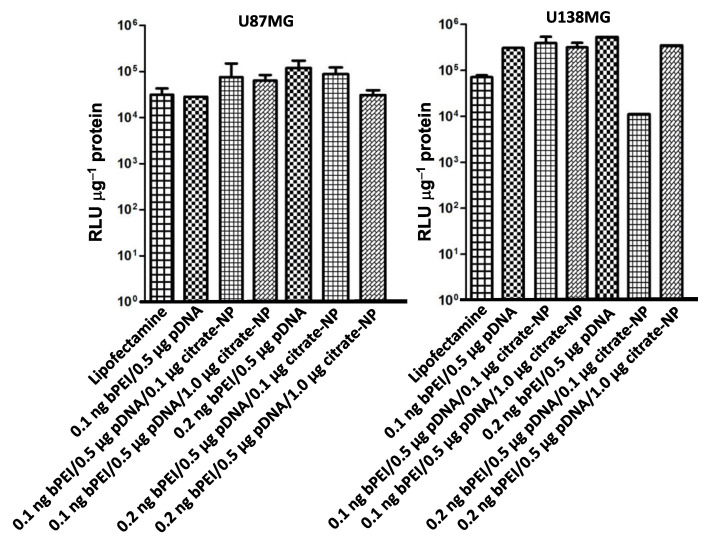
Luciferase expression of different composite transfected to U87MG (left) and U138MG (right) glioblastoma cells for 5 h. The amount of plasmid pRL-CMV pDNA of all composites was fixed at 0.5 μg/well. The amount of lipofectamine was fixed at 2.0 μg/well. *Renilla* activities (RLU) were measured and normalized against total cellular protein per well.

Transfecting U87MG cells with the pRL-CMV pDNA-containing ternary composites resulted in a range of 10^4^ to 10^5^ RLU (Figure 3, left). Generally, higher RLU signal was observed with U138MG cells (Figure 3, right) then U87MG cells. Noticeably, the RLU from the composite 0.2 ng bPEI/0.5 μg pRL-CMV pDNA/0.1 μg citrate-NP in U138MG cells was exceptionally low. However, 0.1 ng bPEI/0.5 μg pRL-CMV pDNA/0.1 μg citrate-NP gave the highest luciferase activity in both glioblastoma cell lines.

**Figure 4:**
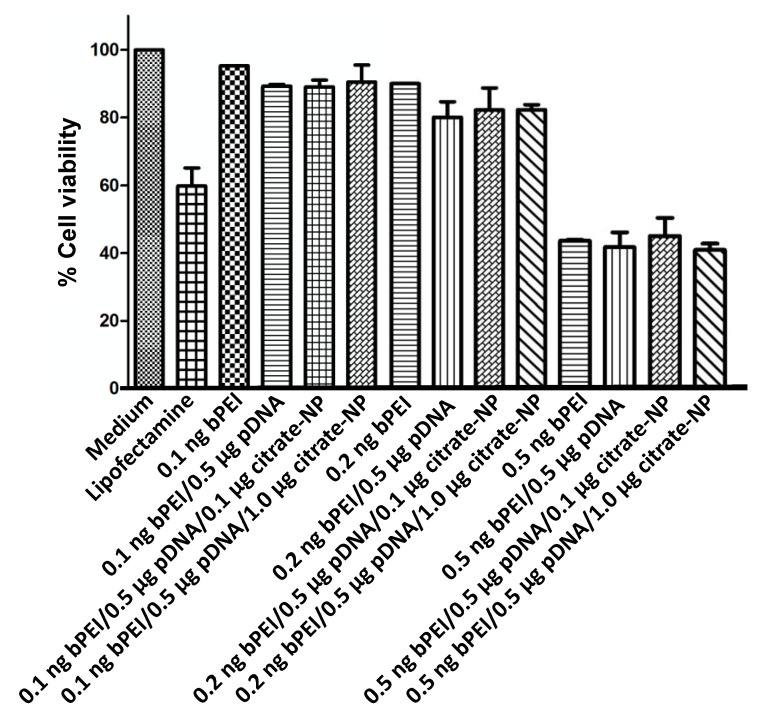
Cell viability of U87MG cells as determined by MTT assay 24 h after incubation with each composite for 5 h. The concentration of plasmid pEGFP-C1 pDNA of all composites was fixed at 0.5 μg/well. The amount of lipofectamine was fixed at 2.0 μg/well.

Ternary magnetic composites and the controls, i.e., medium alone, lipofectamine, branched bPEI alone, and bPEI/pDNA composites, were prepared and that their cytotoxicities in U87MG cells were evaluated by MTT assay (Figure 4). Generally, percentages cell viability decreased with increasing amounts of bPEI from 0.1 ng/well or 0.2 ng (80–90%) to 0.5 ng (40–50%). These results also revealed that the cytotoxicity of using 0.5 ng bPEI was approximately 10% higher than the commercially available transfection agent lipofectamine.

## 4. Discussion

The Infrared absorbance of the citrate-NPs demonstrated that the successful surface modification of iron hydroxyl groups to citrate in the presence of the carbonyl absorption at 1624 cm^−1^. Ternary composites based on different combination amounts of branched bPEI (0.1 or 0.2 ng), citrate-NP (0.1, 1.0, or 2.5 μg), and pEGFP-C1 or pRL-CMV pDNA (0.5 μg) were studied for optimization of the best composite. It is reasonably to consider that the cellular uptake efficiency depends on the surface charge density, stability of the composites and further cleavage of the composite with citrate-NP localized in cytoplasm and pDNA in nucleus. The composites would eventually be dissociated into separate components and after the nanomaterial’s uptaken mechanism. Therefore, it is essential to tune the components’ ratios to study the uptake of NP and nucleic acids towards U87MG and U138MG glioblastoma cells. For the best MRI observations, the use of 1.0 μg/well citrate-NP would be favourable. However, 0.1 ng bPEI/0.5 μg pDNA/0.1 μg citrate-NP would give an optimal gene delivery into the U87MG cells. pDNA pRL-CMV carried a *Renilia* luciferase gene thus it had to be translocated into the nuclei of U87MG or U138MG cells for the gene transcription process. Cellular uptake efficiencies of plasmid pDNA in the ternary composites towards U138MG cells were generally similar to that of U87MG cells. The amount of pDNA that was successfully transfected, was directly proportional to the luminescent signal generated from luciferase expression. Therefore, a composite with a high luciferase activity revealed higher gene delivery efficiency into the glioblastoma cells. Most of the composites had a RLU value higher than that of the commercially available transfecting agent lipofectamine that acted on a positive control in the present study. The ternary composite 0.1 ng bPEI/0.5 μg pDNA/0.1 μg citrate-NP gave the highest luciferase activity and fluorescence signal in both glioblastoma cell lines. The best working bPEI concentration range should not exceed 0.2 ng/well to achieve a relatively low cytotoxicity.

## 5. Conclusions

Different ternary composites, based on branched bPEI (0.1 or 0.2 ng), negatively charged citrate-coated, ultrasmall superparamagnetic iron oxide nanoparticles citrate-NPs (0.1, 1.0, or 2.5 μg), and pEGFP-C1 or pRL-CMV circular plasmid pDNA (0.5 μg), were studied for optimization of the best composite for the uptake into U87MG or U138MG glioblastoma cells. The uptake efficiency in terms of citrate-NP and pDNA gene delivery with the ternary composites could be altered by tuning the bPEI/citrate-NP ratios in the composite, thereby characterized by Prussian blue staining, in vitro MRI as well as GFP and luciferase expression. Among the composites prepared, 0.2 ng bPEI/0.5 μg pDNA/1.0 μg citrate-NP ternary composite possessed the best cellular uptake efficiency of NP evident by MRI assessments and Prussian blue staining. Composite comprising 0.1 ng bPEI/0.5 μg pDNA/0.1 μg citrate-NP gave the optimal efficiency for the cellular uptake of the two circular plasmid pDNAs to the nucleus. The cytotoxicity became significant when 0.5 ng/well of bPEI was present in the ternary composites. The best working bPEI concentration range should not exceed 0.2 ng/well to achieve a relatively low cytotoxicity. As a result, as-prepared polymer composites or other novel nanostructured magnetic composites offered potential biomedical applications in simultaneous gene delivery, imaging contrast enhancement, and mechanistic study. To achieve the next generation in vivo nano-theranostic anti-cancer drug delivery systems, more sophisticated, stimuli-responsive polymeric/dendritic nanostructures held by mechanical bonds for bio-evaluation on imaging and active drug release on demand [40,41,42,43,44,45,46] would be developed for targeting various brain tumours.

## Data Availability

The data presented in this study are available on request from the corresponding authors.
